# Biology of Glioblastoma Multiforme—Exploration of Mitotic Catastrophe as a Potential Treatment Modality

**DOI:** 10.3390/ijms21155324

**Published:** 2020-07-27

**Authors:** Barbora Vitovcova, Veronika Skarkova, Kamil Rudolf, Emil Rudolf

**Affiliations:** Department of Medical Biology and Genetics, Charles University Faculty of Medicine in Hradec Kralove, Zborovska 2089, 500 03 Hradec Kralove, Czech Republic; vitovcob@lfhk.cuni.cz (B.V.); hanusovaV@lfhk.cuni.cz (V.S.); rudolfk@seznam.cz (K.R.)

**Keywords:** glioblastoma multiforme, mitotic catastrophe, microtubule-targeting agents, cell death, benzimidazole carbamates

## Abstract

Glioblastoma multiforme (GBM) represents approximately 60% of all brain tumors in adults. This malignancy shows a high biological and genetic heterogeneity associated with exceptional aggressiveness, leading to a poor survival of patients. This review provides a summary of the basic biology of GBM cells with emphasis on cell cycle and cytoskeletal apparatus of these cells, in particular microtubules. Their involvement in the important oncosuppressive process called mitotic catastrophe will next be discussed along with select examples of microtubule-targeting agents, which are currently explored in this respect such as benzimidazole carbamate compounds. Select microtubule-targeting agents, in particular benzimidazole carbamates, induce G_2_/M cell cycle arrest and mitotic catastrophe in tumor cells including GBM, resulting in phenotypically variable cell fates such as mitotic death or mitotic slippage with subsequent cell demise or permanent arrest leading to senescence. Their effect is coupled with low toxicity in normal cells and not developed chemoresistance. Given the lack of efficient cytostatics or modern molecular target-specific compounds in the treatment of GBM, drugs inducing mitotic catastrophe might offer a new, efficient alternative to the existing clinical management of this at present incurable malignancy.

## 1. Introduction

Malignant tumors of the central nervous system (CNS) comprise both cases arising mostly in the brain and to a minor extent in other parts of the CNS as well as metastatic malignancies originating from other tissues and/or anatomical parts in the body. In the former group of conditions, the most frequently occurring are malignant gliomas (accounting for up to 80% of adult brain tumors), which are traditionally categorized according to their cellular origin, histopathological features and clinical manifestation. Using these criteria, World Health Organization classifies gliomas into four groups-grades, with each of them reflecting the level of malignant phenotypes associated with glioma cells. Typically, grade I gliomas are largely viewed as benign with relatively good patient prognosis if it is possible to remove the tumor mass surgically, while higher-grade gliomas show increasingly pathological features and behavior, resulting in their diffuse spread throughout the brain, resistance to therapy and incurred damage to the brain tissues, leading to ultimate and rather fast patient lethality [1]. The most malignant and aggressive type of glioma, i.e., grade IV glioma, is termed glioblastoma multiforme (GBM), which represents approximately 60% of all brain tumors in adults. Despite the fact that GBM global incidence is considered statistically low (5–10 cases per 100,000 people), its biological and genetic heterogeneity combined with exceptional aggressiveness and very ineffective available therapies result in poor prognosis for patients whose survival rate even upon the best clinical management rarely exceeds 15 months following the initial diagnosis [2].

In this review, the basic biology of GBM cells is summarized with emphasis on cell cycle and cytoskeletal apparatus of these cells, in particular microtubules. Their involvement in the important oncosuppressive process called mitotic catastrophe will next be explored. Given the lack of efficient cytostatics or modern molecular target-specific compounds in the treatment of GBM owing to their limited access via hematoencephalic barrier and/or due to the intrinsic or acquired resistance of malignant astrocytes, drugs inducing mitotic catastrophe might offer a new, efficient alternative to the existing clinical management of this at present incurable malignancy.

## 2. Molecular Classification of GBM

Technological advancements in molecular diagnostics and, in particular, use of gene expression profiling, have been instrumental in our understanding of GBM diversity, leading to the identification of its four major subtypes (i.e., proneural, neural, classical, and mesenchymal) and ultimately helped in determination of the origin of this malignancy [3]. Thus nowadays we recognize primary GBMs arising de novo, predominantly in elderly patients (up to 90% of GBM cases), and secondary GBMs developing from preexisting lower-grade gliomas and often diagnosed in younger patients (10% of GBM cases). One characteristic molecular difference between primary and secondary GBMs is the mutational state of isocitrate dehydrogenase (*IDH*) genes, with *IDH* wild-type being present most frequently in the primary GBM, whereas *IDH* mutant type associates more commonly with the secondary GBM [1]. Both GBM types next harbor several typical genetic alterations in key genes regulating growth factors, cell cycle regulators, DNA repair, survival and cell migration, with corresponding associated upregulated or downregulated signaling pathways [4,5]. In addition, a number of less explored genetic changes such as copy number alterations in other genes on the corresponding chromosomes have been identified in individual GBM types alongside differences in DNA methylation [6], histone acetylation and expression of non-coding RNAs [7]. Such evidence increasingly contributes to the more specific typing of individuals diagnosed GBMs and enables detailed appreciation of often less robust molecular signatures hitherto not acknowledged, not only in newly diagnosed cases but also in recurrent tumors. Accordingly, it may be expected that new types of GBMs will be identified in the future based on unique molecular changes as proposed recently [4,5]. Finally, since the detailed description of genetic and epigenetic changes in GBM was not the primary focus of the present work, interested readers will find more in-depth information in several other published reports [8,9,10].

## 3. Cytoskeleton of Astrocytes and Malignant GBM Cells

Primary GBM is characterized by both diffuse infiltration and invasion of residual tumor cells, which also explains the high recurrence rate of this malignancy collectively leading to early patient lethality. This feature clearly implicates the cytoskeleton as a key cellular compartment modulating such behavior.

Today, we know that the cellular cytoskeleton is responsible for a wide array of functions in both cellular and tissue contexts. These span mechanical stabilization of cells’ shape, size and adherence; intercellular connections; and various modes of communications within the cell, between individual cells or between cell(s) and the surrounding environment. Many if not most cell-autonomous processes such as cell division, migration, gene expression, intracellular transport, differentiation, metabolism or signaling depend on the cytoskeleton. The major reason for such intricate involvement of the cytoskeleton is its structure and diversity, with the three basic types of fibers (e.g., microtubules—MTs, microfilaments—MF—and intermediate filaments—IFs) present in all cells together with a number of associated proteins and other modulating molecules [11].

Astrocytes originate from radial glial cells, and through a series of steps they mature and migrate to the designated position in the brain [12]. There they begin to assume their final spongy stellate morphology, which involves, among other things, extensive changes in their cytoskeleton. These include dense packing of MTs and their accumulation in the main cellular processes and remodeling of contractile actin fibers in favor of Arp2/3-dependent branched actin arrays [13] with associated shifts in corresponding regulatory signaling pathways, i.e., inhibition of Rock-RhoA axis and activation of Rac1 [14]. Similar to MTs, IFs localize mostly into astrocytic processes of mature cells, but unlike MTs and MFs, they show differential expression at different stages of development. Thus non-mature astrocytes are positive for vimentin and synemin, while mature astrocytes express glial fibrillary acidic protein (GFAP) and vimentin [15].

Malignant transformation represents a complex process of general reprogramming of the target cell into oncogenic phenotype, which involves extensive changes in cytoskeleton too. In the case of GBM cells, the reported changes entail all cytoskeletal elements and their regulation. Still, at present, the dynamics of these changes are not thoroughly mapped since they firstly occur in a cell-autonomous manner, but at later stages, they are no doubt significantly influenced by tumor microenvironment, in particular hypoxia [16]. Thus the following list of examples of cytoskeletal alterations in malignant glioma cells does not faithfully recapitulate the entire progress of GBM development nor distinguish between heterogeneous cell clones present in this tumor.

One of the hallmarks of GBM cells (at least some) is their highly motile and infiltrative nature. GBM cells have been generally described to spread to the surrounding brain tissues using the perivascular space around blood vessels and axons [17]; however, details concerning their selection algorithms for different routes as much as the existence of other strategies are still not fully understood. Using various types of cultures and fluorescent imaging, several research groups have demonstrated that malignant cells in GBM migrate individually, using the mesenchymal mode of cell migration and invasion [18,19,20]. Molecularly, this motile activity is associated with changes in cell polarity, actin polymerization and organization (see above) and results from differential expression and activity of small Rho GTPases Rac1, Cdc42, Rho and their targets [21,22,23], although their mutual interactions are still a matter of intensive scientific inquiry. Many studies have also provided evidence on upregulation of several MF-associated proteins, which include plasma membrane and MF-linking moesin and ezrin [24,25,26] and MF-organizing profilin, filamin, fascin and others [24,27,28,29]. However, GBM cells may be more flexible in the selection of the mode of their migration and invasion. This context-dependent flexibility is clearly inferred from several lines of evidence. Firstly, the malignant glioma cell membrane is capable of generating blebs for cell protrusion [30,31,32]. Secondly, heterogeneous phenotypes of invasive cells (mesenchymal- and amoeboid-like) may coexist in GBM, with the ongoing cells morphological transitions following different enzymatic interactions with the surrounding matrix. Such plasticity of malignant cells has been demonstrated by Koh et al. [33] with the help of tumor spheres directly established from fresh GBM tissues and via patient-derived GBM cells in three-dimensional tumor model established from decellularized tissue-derived ECM.

Successful infiltration and invasion require the special reprogramming of cells, which is in malignancies arising from epithelia termed as epithelial-to-mesenchymal transition (EMT). Although GBM belongs to nonepithelial tumors, to a certain extent, EMT-like processes may be involved [34]. It is also obvious when another feature of EMT, i.e., ability of cells to remodel ECM via secretion of specific enzymes (mostly matrix metalloproteases—MMPs) at the leading edge to create free corridors, is considered. GBM cell lines and GBM biopsies have been shown to express elevated levels of several MMPs (MMP-1, -2, -7, -9, -11, -12, -14, -15 and -25) as compared to low-grade astrocytomas, although individual samples varied in their expression. On the other hand, authors of this observation cautioned about incompletely understood roles of MMPs in this process as well as about analyses carried out on primary glioblastoma cells as MMP expression might significantly differ under cell culture conditions, and their expression patterns do not correlate well with those obtained from the original GBM patient tumor tissue [35].

Unlike MFs and MTs, IFs show strict cell-type and differential stage-dependent expression, and that is why they are used as identification markers. Here, in contrast to astrocytes, GBM cells may express vimentin and GFAP but also nestin, synemin and other types of Ifs, but their individual subcellular topography and expression levels may considerably vary [36] with unclear impact on select aspects of GBM biology [37,38,39]. In some astrocytomas, for instance, GFAP accumulates and forms distinct aggregates called Rosenthal fibers [40]. In addition, the presence and the abundance of individual IFs in GBM cells correlates more (vimentin and nestin) or less (GFAP) with patient prognosis or tumor staging [41,42].

Similar to the case of MFs and IFs, immunohistochemical analyses and various functional microarrays revealed that malignant GBM cells also vary in the expression of α-, β- and γ-tubulin. In addition, their expressed isoforms and post-translational modifications appear unique for each tumor [43]. Using three standard (U87, U251, A172) and 12 newly established glioblastoma stem cell lines, it was next shown that decreased α/β-tubulin expression is associated with lack of cell differentiation. Moreover, GBM cell sensitivity to microtubule targeting agents (MTAs) is independent of tubulin isotypes and the select post-translational modifications, although the higher levels of α- and β-tubulin generally increase such sensitivity [43]. Besides the mentioned changes, GBM cells contain elevated levels of the unique β-tubulin isotypes, i.e., β-IV and β-III [44,45], where β-III tubulin expression, in particular, has attracted considerable scientific attention since it seemingly correlates with resistance of these cells to microtubule-stabilizing cytostatic drug taxol [46]. Other documented changes concerning γ-tubulin expression in GBM cells may relate to a number of pathological cellular events such as abnormal assembly of mitotic spindle with the subsequent aberrant chromosome segregation leading to genetic instability [47]. To this extent, in astrocytoma cells, γ-tubulin was described to be in a soluble form as compared to centriolar localization in normal cells [48]. Moreover, since γ-tubulin is an essential player in MT nucleation and overall dynamics, altered expression of this protein may also influence overall cell stability and transport dynamics as well as other processes such as cell adhesion, polarity and motility. The role of MTs, and, in particular, MTs in GBM cells in modulation of cell motility, is further documented by tumor-specific changes in several microtubule-associated proteins (MAPs) involved in cell locomotion. These include an astrocytoma-splice variant MAP-2e concentrated at the invasive front of the tumors [49], doublecortin [50], RHAMM/IHABP [51], dynein, spastin and others [52,53].

MTs in malignant cells were among the earliest identified and employed targets of cytostatic therapy. In GBM, the use of traditional destabilizing (for instance vinca alkaloids) and stabilizing (taxanes) MTAs proved to be inefficient largely due to restricted access of these agents via hemato-encephalic barrier and existing chemoresistance of tumor cells. Thus, new MT-associated targets along with newly discovered or developed compounds are being studied, as it is still believed that this cellular compartment might be an important key in the treatment of GBM [46,54]. In this respect, one novel approach in this area is the exploration of the process called mitotic catastrophe, which may be triggered by MTAs alone or in combination with other therapeutics.

## 4. Cell Cycle

The ability of eukaryotic cells to reproduce by the process of cell division relates to a series of events which are known under the collective term cell cycle. The main purpose of cell cycle is to ensure accurate DNA replication (S phase) and final generation of two identical daughter cells (M phase). To maintain optimal cell cycle progress, cells employ a variety of mechanisms including several control points. These integrate various external and internal inputs through a complex system of positive and negative feedbacks to allow or to retard transitions between individual cell cycle stages. Together, there are three major control points or checkpoints recognized in eukaryotic cells; the first occurring near the end of the G_1_ phase, the second at the G_2_/M phase transition and the third (also called spindle assembly checkpoint—SAC) placed at the metaphase-to-anaphase transition. The G_1_ checkpoint controls appropriate cell growth and environmental stimulation as well as checks DNA integrity. The central coordinator of this checkpoint signaling is Retinoblastoma (Rb) protein, whose activity is regulated via its interaction with a number of molecules including p16, cyclin-dependent kinases 4 and 6 (CDK4/6), cyclin D and a family of transcription factors E2F. Moreover, in response to detected DNA damage, various sensory (ataxia-teleangiectasia-mutated—ATM or ataxia-teleangiectasia-Rad3-related—ATR) and effector (checkpoint kinase 1 or 2–Chk1 or Chk2) kinases are recruited along with p53 protein, which mediates cell cycle arrest via CDK-inhibiting protein p21 to allow for DNA repair or to stimulate senescence/cell death [55]. Similarly, the G2/M checkpoint aims to prevent entry of the cell to mitosis in the presence of DNA damage with ATM kinases, dual-specificity phosphatase Cdc25, p53 protein and M phase cyclin (CDK) complexes involved [56]. Finally, the SAC senses bipolar tension generated by appropriate alignment of chromosomes at the metaphase plate, which results in degradation of cyclin B via anaphase-promoting complex APC/C and beginning of mitotic exit and chromatid separation [57]. The activity, coordination and timing of all the mentioned checkpoints thus governs the progress of the cell throughout the cell cycle upon the appropriate environmental context, and any defects in these control mechanisms predispose to aberrant cell cycling, which is regularly seen with tumor cells.

Many analyses of genomic, proteomic and cellomic landscapes of GBM revealed and confirmed significant intratumoral heterogeneity on many levels with a characteristic temporal and spatial development [58,59]. This heterogeneity also entails the coexistence within a GBM tumor mass of distinct cell populations with individual cell cycle status related to the particular cell cycle regulating or influencing molecules. Accordingly, malignant GBM cells have been reported to harbor multiple genetic abnormalities leading to deregulation of cell cycle via defunct checkpoints. Specifically, in about 50% of GBM cases, p16 was reported to be deleted or, alternatively, silenced by methylation [60]. Similarly, the expression of Rb protein may frequently be absent in GBM cells too [61]. The prevalence of abnormal regulation of p16/Rb signaling in this malignancy is further underscored by the fact that both p16 and Rb abnormalities seem to be mutually exclusive [61,62]. Lastly, E2F-1 transcription factor was found to be significantly upregulated in T98, U251, U138, U87, U343, M059J and M059K GBM cell lines [63], thereby pointing to the importance of deregulated Rb/p16/E2F axis in this malignancy. This conclusion is further corroborated by the observations that G_1_ checkpoint positive regulator proteins cyclin D1 and CDK4/6 are overexpressed in GBM [9].

Mutations and other changes in protein p53 and its dependent pathways as part of both G_1_ and G_2_/M checkpoint signaling have also been detected in GBM, although their frequency and importance for development and maintenance of malignant phenotype differ in relation to the GBM type [64,65].

Various chromosomal aberrations seen in advanced malignancies including GBM suggest their likely origin from aberrant mitoses [66]. This in turn points to a possible presence of defects in the SAC signaling and activity. To this end, genetic studies carried out on patient-derived GBM stem-like cells revealed defective kinetochore microtubule attachments associated with BUB1B/BubR1 and BuGZ genes. These genes seem to be primed by oncogenic signaling in GBM, which enables malignant cells to tolerate abnormalities in chromosome alignment and separation [67,68]. Aberrant SAC and resulting cytogenetic heterogeneity malignant cells may, on the other hand, be explored as a potential therapeutic target. In this context, it has been proposed that any further disturbance or disruption of mitotic spindle and related SAC activity might exceed the threshold of tolerance in malignant cells, thereby driving their rapid and thorough demise [69].

## 5. Checkpoint Inhibition

Similar to other highly malignant solid tumors, GBM cells show aberrant cycling and increased proliferation, which is associated with deregulated checkpoints as outlined above. Accordingly, these behaviors started to be exploited as a therapeutic target once the basic principles of chemo- and radiotherapy were established [70]. Thus, until today, the specific treatments in this field aim to interfere with (1) cellular components and events linked with cell cycle and cell division such as DNA integrity and replication, mitotic spindle activity and contractile ring formation and function or target; and (2) individual molecules regulating cell cycle progress and cell division as reviewed in [71]. The mechanism of action of many traditional (i.e., MTAs) as well as newer targeted agents (i.e., CDK, aurora kinase or polo-like kinase inhibitors) involves G_2_/M inhibition [71]. This approach presents several advantages. Firstly, despite ongoing discovery of new classes of antineoplastics, many traditional compounds (i.e., MTAs) continue to be standards in curative and palliative oncological care [72]. Secondly, these agents may synergize with the current standards in GBM therapy, i.e., temozolomide or radiation, and enhance their DNA damaging effects or sensitize malignant cells to them [73,74]. Thirdly, since many of these compounds interfere with mitosis, they may enhance instability of mitosis-emerging cells to ultimately bring their demise via the process of mitotic catastrophe.

## 6. Mitotic Catastrophe

**Figure 1:**
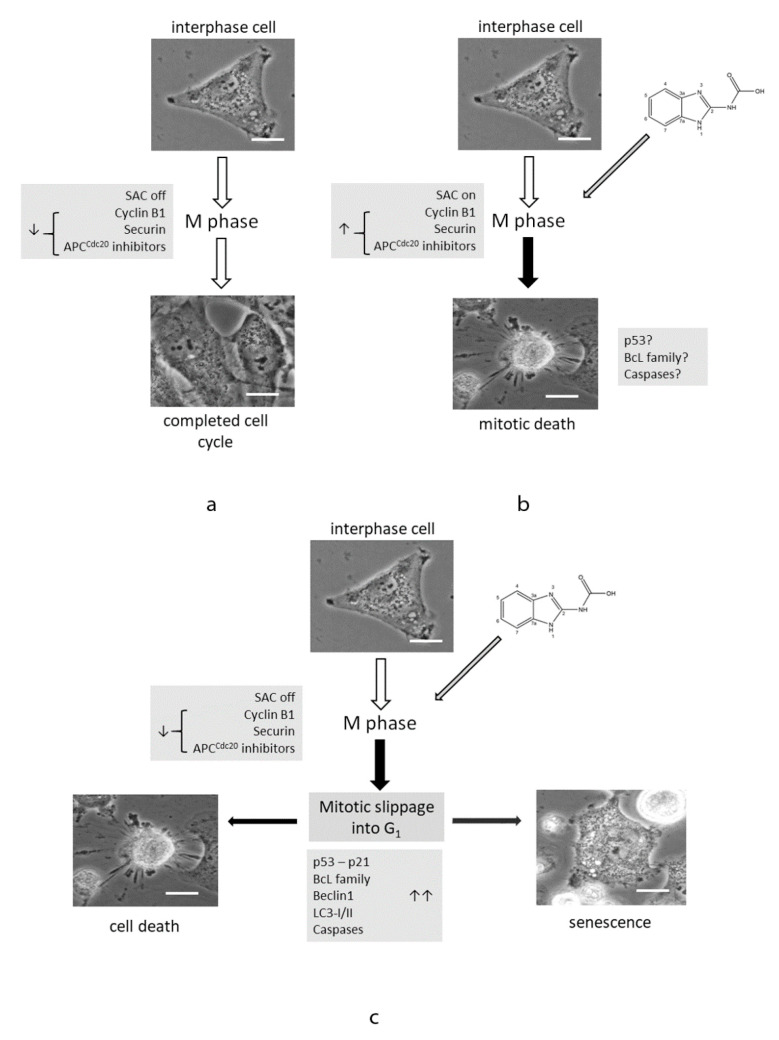
Modes of glioblastoma (GBM) cells responses to benzimidazole carbamates. (**a**) During the normal course of mitosis, spindle assembly checkpoint (SAC) is inactivated by sensing productive attachments of chromosomal kinetochores to spindle microtubules. Anaphase promoting complex inhibitors APC^Cdc20^ decay, which is associated with securin degradation and separase-mediated cleavage of cohesin with subsequent chromatid separation. Finally, cyclin B1 degradation inactivates cyclin-dependent kinase 1 (CDK1), which allows the cell to exit mitosis, thereby completing cell cycle and division [84]. (**b**) Microtubule targeting agents (MTAs) such as benzimidazole carbamates may activate the cell death program while the cell persists in mitosis in the presence of activated SAC and elevated levels of cyclin B1. This mitotic death may proceed via activated p53-dependent apoptotic signaling including select BcL-family proteins and in the presence/absence of activated caspases. (**c**) Treatment with MTAs such as benzimidazole carbamates may also lead to activation of stress pathways, which nevertheless allow the cell to escape mitosis (so-called mitotic slippage). The final cell fate then depends on the extent and nature of activated signaling, which may lead to the immediate or delayed cell death, again with involvement of p53-dependent signaling and BcL family proteins. Alternatively, cells may upregulate autophagy via increased expression of Beclin-1 or enter an irreversible cell cycle arrest—senescence, possibly via p53-p21 signaling axis [85]. Scale bar 5 µm, phase contrast 600×.

In the past decades, the term mitotic catastrophe (MC) has been used to describe a mode of cell death resulting from abnormal mitosis caused by diverse stressors. Our current understanding of this process embraces its more functional definition as it is postulated that MC represents a sequence of events which acts to prevent genomic instability of cells via inducing mitosis-linked delayed cell death or permanent cell cycle arrest with subsequent senescence. As such, under physiological circumstances, MC functions as one of the oncosuppressive mechanisms which has recently gained considerable interest among biomedical scientists due to its potential to eliminate potential or nascent tumor cells. Cells where MC is stimulated often increase their volume, show the gross nuclear alterations such as micro- and macronucleation and may accumulate in the particular phase of the cell cycle. Still, since the process of MC and the type of (malignant) cell, as well as the nature of triggering stimulus, are not homogeneous, the activated signaling and the final cell phenotype are more than likely to differ. Accordingly, at least three scenarios of MC have been described [69,75] in which (1) the cell might activate cell death machinery in the presence of elevated cyclin B1 levels, i.e., while it is still in mitosis, or (2) the cell is firstly allowed to complete mitosis and in the subsequent interphase may undergo cell death, in a delayed manner. This particular instance is referred to as mitotic slippage or mitotic checkpoint adaptation. Finally, (3) the cell is firstly allowed to complete mitosis and in the subsequent interphase develops the senescent phenotype [76]. Accordingly, tumor cell populations exposed to MC-inducing agents are likely to respond with a significant degree of heterogeneity including the appearance of several cell phenotypes whose relative proportions and fates may ultimately reflect the evolutionary status and the nature of the particular tumor [77,78,79]. Thus several research groups have reported that tumor cells exposed to benzimidazoles in vitro become polyploid, aneuploid or senescent, upregulate autophagy and die via apoptosis or necrosis [80,81,82]. In Figure 1, two possible scenarios of benzimidazole-exposed GBM cells responses are shown including cell morphologies as well as select molecular players. Morphological appearance of cells exposed to MC-inducing agents provides basic clues about the spectrum and rates of individual cell phenotypes (i.e.,); however, as such, is considered inadequate without the molecular nature of the underlying processes [83]. Accordingly, additional or alternative verification of cell status is necessary due to at least two reasons. Firstly, diagnostic procedures in clinical practice rely mostly on histopathological examination of the preserved tissue/tumor section where an overall architecture is graded, while nowadays the emphasis on the expression of a given molecular marker too. The direct evaluation of cell phenotypes is thus limited to in vitro or ex vivo studies.

Secondly, molecular pathways that regulate MC and determine the final mode of cell response are still not completely understood. Generally speaking, diverse factors may trigger MC, including DNA damage, checkpoint inhibition and general stress (i.e., hyperthermia), as well as mitosis-addressing agents (i.e., MTAs or small molecule inhibitors) [86]. MTAs induce MC by their interference with mitotic spindle, which leads to perturbations in spindle assembly checkpoint (SAC), incorrect segregation of chromosomes and activation of the corresponding signaling. This signaling may include the activation of protein p53 and its dependent circuits, Bcl-2 family proteins and various execution substrates (i.e., caspases) whose individual wiring determines the cellular endpoints [83,87,88]. Moreover, some studies carried out on cancer cell models demonstrated that mitochondrial-targeted proteins Mcl-1 and Bcl-xL orchestrate MC duration, which, in turn, determines the interplay between MC-activated autophagy and cell death [89]. This particular discovery is very interesting because if thus activated, autophagy could lead to cell demise, and it may succeed even in tumor cells defective for apoptotic regulation [90]. In this context, it remains to be seen whether the reported autophagy leading to cell death following drug-dependent MC activation is a type of general response of tumor cells or, rather, the specific circumstance limited to the unique malignant cell population challenged by the concrete MC stimulus.

MC and its role in suppression of GBM cells have not been intensively researched so far. Moreover, since MTAs have shown negligible utility in the clinical therapeutic regimens of GBMs due to the above-discussed limitations, there are very few studies where the MTAs potential to induce MC in GBM cells has been addressed. One exception is noscapine, a phthalide isoquinoline alkaloid from the plant of genus Papaver that binds β-tubulin at a unique site and alters its conformation, leading to a stalled microtubule state and resulting in mitotic arrest. Noscapine has been studied with several in vitro and in vivo experimental models; it showed antiproliferative and cell-death-promoting effects in C6 rat glioma cell line [91], and it reduced clonogenic potential of human T98G and murine GL261 glioma cell lines while exerting low toxicity in normal astrocytes [92,93]. It was also efficient in suppressing the growth and inducing mitochondrial apoptosis in four human glioma cell lines [94]. Similar efficiency was noted in noscapine-treated A172, LN229 and U251 GBM cells with established resistance against temozolomide [95]. Finally, it significantly increased survival of animals intracranially inoculated with temozolomide-resistant GBM cells [95]. Noscapine also proved to enhance toxicity of other cytostatics such as temozolomide, BCNU or cisplatin and radiation therapy [95,96,97,98]. Thus, noscapine-specific effects towards GBN cells seem very promising but its further studies might be limited due to its relatively short half-life and poor solubility. To overcome these limits, several noscapine derivates were prepared and subsequently tested on GBM and other malignant tumor models with positive results [99,100,101,102,103].

**Figure 2:**
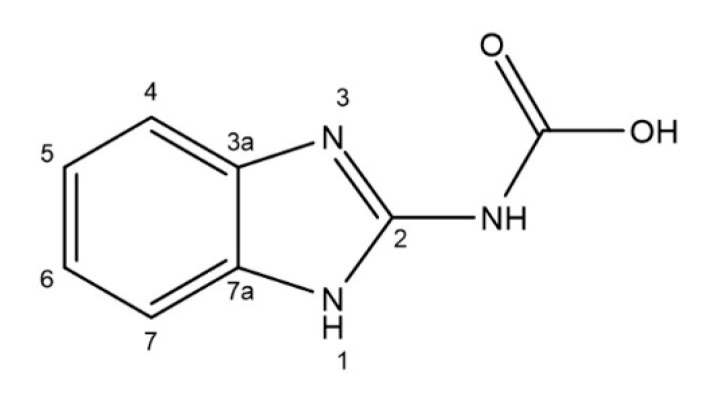
The structure of benzimidazole carbamate molecule.

Benzimidazole carbamates (Figure 2) are compounds approved as anthelminthics in human and veterinary medicine. This group includes several members such as mebendazole, albendazole, fenbendazole or flubendazole, which exert their effects against worms via binding and inhibiting β-tubulin [104,105].

Given their mechanism of action, these compounds were “repurposed”, i.e., tested in a different indication area—oncology—as potential antineoplastic agents and proved to be preclinically efficient in many types of malignant tumors [106,107,108,109,110]. In human GBM cells U87-MG (U87), D54, H80, H247, H392, H397, H502, H566 and the mouse GL261 glioma cell line, mebendazole demonstrated cytotoxicity with low IC_50_ values. Mebendazole reduced microtubule polymerization in exposed GBM cells and significantly extended mean survival in syngeneic and xenograft orthotopic mouse glioma models [111]. Based on these results, a clinical trial with the aim of finding the highest dose of mebendazole that can be safely given to people with high-grade glioma in combination with the current standard of care (temozolomide) without causing severe side effects was started in April 2013 with the nowadays set primary completion in September 2016 and estimated study completion in September 2025. In this intervention single-group study, mebendazole will be given to patients three times every day orally with meals on a 28-day cycle. Apart from its primary objective; i.e., to determine the maximum tolerated dose of mebendazole in combination with temozolomide (TMZ) given after surgery and the standard radiation and TMZ treatment in patients with newly diagnosed malignant gliomas, the overall patients’ survival (10-year frame) will be measured (https://clinicaltrials.gov/ct2/show/NCT01729260). Another member of the benzimidazole family, flubendazole, has been found effective against two human glioma cell lines SF-268 and T-98G, in which it induced G_2_/M cell cycle arrest, upregulated p53 expression and reduced cyclin B1 and p-cdc2 expression. This activity led to cell apoptosis via downregulation of Bcl-2 expression. Flubendazole also successfully suppressed the growth of glioma xenograft models in mouse [82].

## 7. Conclusions and Future Outlook

Despite concerted scientific efforts and accumulation of experimental and clinical data about the biological nature and behavior of GBM, this type of malignancy remains largely incurable, with the currently used therapeutic regimens being of limited value. It is thus more than necessary to use new approaches and exploit GBM specific features to our advantage to bring this type of malignancy under control. The ultimate goal of our efforts should be, if not prevention of GBM development, then its successful physical or functional elimination. This review provided ample evidence on the complexity of GBM origin, development, and behavior, which do reflect the complicated terrain where we aspire to interfere. Conversely, a number of unique features of GBM cells, namely the extent and specificity of cytoskeletal (microtubular) reprogramming, offer an attractive target of possible intervention. Although the classical MTAs proved to be largely ineffective both as single agents or in combined regimens of GBM treatment, scientific interest in finding other cytoskeleton-specific targets in malignant glioma cells continues as evidenced in several recent reviews. MC is nowadays viewed as a way of elimination of genomically unstable cells via diverse cellular endpoint phenotypes and as such represents an attractive platform for the development of novel antineoplastic agents. Viability of this concept is demonstrated by the fact that many malignant cells, including GBM, are heteroploid and thus intrinsically prone to the aberrant course of mitosis, activation of MC and their elimination. In addition, MC in target cells may be induced with considerably lower concentrations of employed agents, which is very beneficial due to the reduction of side-effects-related toxicity. Finally, MC may be successfully employed as an additional effect of combined therapies, which would maximize the clinical efficiency upon minimized toxicities or off-target effects. This point is strongly supported by the current state of MC exploration in GBM, where relatively few reported studies (59 hits in PubMed–December 2019) often investigated this phenomenon in relation to combined effects of radiation or temozolomide with other sensitizing agents. In this respect, several discussed MTAs known to induce MC in GBM cells seem promising, but their future potential and application in treatment protocols will most likely be in chemotherapy or radiotherapy sensitization.
